# Standard practices in cardiac monitoring: training needs of intensive care unit nurses

**DOI:** 10.1186/s12912-024-01742-1

**Published:** 2024-01-31

**Authors:** Angela Carolina B. de S. Giusti, Marilia Estevam Cornélio, Elaine Machado de Oliveira, Jean-François Giguère, Maria Cecília B. J. Gallani

**Affiliations:** 1 School of Nursing, University of Campinas, Campinas, São Paulo, Brazil; 2 CHU de Québec-Université Laval, Quebec, Canada; 3 Faculté des sciences infirmière - Université Laval, Quebec, Canada

**Keywords:** Intensive care units, Nurses, Education, Needs Assessment, Monitoring, Arrhythmias

## Abstract

**Background:**

Enforcing practice standards for cardiac monitoring in intensive care units (ICUs) has been shown to reduce misdiagnoses and inappropriate interventions. Continuous professional development (CPD) programs are committed to aligning clinical practices with recommended standards. The crucial initial phase in CPD development involves assessing the training needs of the targeted population.

**Objective:**

To assess the training needs of ICU nurses in cardiac monitoring. The overarching goal was to formulate a focused Continuous Professional Development (CPD) program geared towards implementing standard practices in cardiac monitoring.

**Methods:**

This study employed a generic qualitative approach with a descriptive design, utilizing interviews and focus groups from July to September 2018. Involving 16 ICU nurses. Content analysis was employed, encompassing transcription, fluctuant and iterative reading, unitization, categorization, coding, description, and interpretation.

**Results:**

All nurses recognized cardiac monitoring’s importance in the ICU but reported barriers to its effective implementation which were related to factors that could addressed by a CPD as insufficient knowledge and skills. Training needs were identified in both clinical and technical aspects, with recommendations for practical and theoretical activities and e-learning strategies. Barriers related to organizational aspects (equipment and communication within the healthcare team) were also mentioned.

**Conclusion:**

ICU nurses presented clear and specific training needs related to cardiac monitoring as knowledge, skills, and competencies. Other organizational aspects were also reported as barriers. Addressing these learning needs through targeted CPD aligned with organizational initiatives can contribute to enhancing the quality of cardiac monitoring practices in ICUs.

**Supplementary Information:**

The online version contains supplementary material available at 10.1186/s12912-024-01742-1.

## Introduction

Introduced in hospital units 60 years ago, cardiac monitoring has undergone significant evolution propelled by technological advancements. This evolution has expanded its goals beyond simple heart rate detection now encompassing detection and recording of complex arrhythmias, identification of conditions leading to fatal arrhythmias like prolonged QT intervals and playing a crucial role in detecting myocardial ischemia [1–3].

Early identification of cardiac rhythm disorders improves patient care and safety outcomes [4–5]. However, the last American Heart Association guideline underscores the persistent issue of inappropriate use or underuse of cardiac monitoring in Intensive Care Unit (ICU) settings revealing a striking inconsistency between scientific recommendations and their application in clinical practice [6].

Nurses play a vital role in utilizing cardiac monitoring within the ICU setting, bearing responsibility for both technical aspects of monitoring and the clinical judgment and decision-making upon data interpretation [7–9]. Unfortunately, studies indicate a prevalent misuse and underutilization of this technology by nurses [4, 6, 7] diminishing its potential to guide clinical decision-making [7–11] that can be explained partially by their lack of essential knowledge and expertise in cardiac monitoring [10–11]. Common problems in monitoring include improper electrode positioning, inadequate skin preparation, failure to document and notate electrocardiographic changes, and lack awareness about indications for cardiac monitoring. However, challenges extent beyond technical concerns, encompassing overuse of arrhythmia monitoring, underuse of QT interval and ST-segment monitoring in specific patients’ groups, alarm fatigue, misdiagnoses, and poor documentation– all contributing to negative impacts on patient outcomes [6, 12–14]. Additionally, nurses express a lack of confidence in identifying and interpreting electrocardiographic rhythms, thus impacting their ability to provide optimal interventions [15–16]. These issues are directly linked to patient morbidity and mortality [17–18].

In 2004, the American Heart Association (AHA) Councils on Cardiovascular Nursing, Clinical Cardiology, Cardiovascular Diseases in the Young, and the International Society of Computerized Electrocardiology collaborated to establish a consensus on standard practices in cardiac monitoring, aiming to enhance the overall quality of care [12]. This consensus emphasizes the importance of professional training programs to ensure the adoption of optimal practices in cardiac monitoring. These programs should cover competencies, reflecting practical skills related to monitoring settings, the recognition of changes in the electrocardiogram (including arrhythmias and alterations in ST and QT segment morphology), as well as aspects related to event interpretation, communication, and documentation [3–4].

An educational initiative with higher likelihood of enhancing the integration of the standard recommendations into the clinical practice with long-term knowledge retention, enhanced ability to incorporate acquired knowledge into daily routines, is typically embodied in the strategy of Continuous Professional Development (CPD). CPD is a unique human resource development strategy aimed at facilitating workforce readiness, adaptation to technological advancements, and ongoing knowledge enhancement. In the context of cardiac monitoring, this commitment translates to improved patient outcomes.

Blais & Hallée (2003) [19] underscore the significance of actively involving staff members who are the focus of Continuous Professional Development (CPD) training through the training needs elicitation process. This approach aims to shape a more effective assessment, maximize training outcomes, and minimize resistance to change.

Barriers frequently arise from assessments of training needs and play a pivotal role in creating the identified gaps between current reality and desired outcomes. It is imperative to pinpoint barriers that a CPD can address. Barriers related to organizational structure are also important and require attention, but through different interventions, such as administrative decisions. Effectively addressing CPD training needs alongside organizational issues facilitates bridging the gap between expectations and the current reality.

Blais & Hallée (2003) [19] emphasize the critical role of active involvement of staff members throughout the entire CPD training process, placing particular emphasis on the early planning phases that commence with the identification of training needs. This inclusive approach is designed to enhance the effectiveness of assessments, optimize training results, and minimize resistance to change. Recognizing that barriers often emerge during the assessment of training needs; these barriers play a pivotal role in illuminating the gaps between the current state and desired outcomes. While CPD can effectively address several barriers it is crucial to note that barriers associated with organizational structure may also hold significance, and require distinct interventions, such as administrative decisions. In pursuit of translating knowledge and skills into effective clinical practice, it becomes paramount to simultaneously focus on addressing both CPD training needs and organizational challenges. This integrated strategy serves as a deliberate approach to bridge the gap between anticipated expectations and the existing reality. The overarching objective of this comprehensive strategy is to facilitate a smoother integration of training initiatives within the organizational framework, ultimately fostering a more cohesive and effective clinical practice.

Recognizing that (1) The assessment of training needs is essential for the development of an effective CPD program; and (2) An intentional and critical analysis of experience related to the situation gaps allows to identify barriers; [20], the main objective of this qualitative study was to identify the training needs of ICU nurses in cardiac monitoring, through the exploration of their experiences, identification of barriers hindering the implementation of standard practices, and an understanding of their preferences in cardiac monitoring training. The overarching goal of the study was to obtain subsides for supporting the proposal of a targeted CPD aimed at facilitating the adoption of standard practices in cardiac monitoring by ICU nurses.

## Methods

### Design

This study used a generic qualitative design with a qualitative description approach (Kahlke, 2014) [21]. According to Sandelowski (2000) [22] a qualitative description is defined as research designed to produce a low inference description of a phenomenon. The qualitative description attempts to minimize inferences made in order to remain “closer” to the original data. The steps of data collection were guided by the COREQ check list [23] (see Annexes).

This study, carried out from July 2018 to September 2018, marks the initial step of a broader research project dedicated to producing a targeted CPD. However, faced to the challenging circumstances posed by the COVID-19 pandemic on research resources, there was a prioritization of advancing the design of the CPD before releasing the results of the initial study phase.

### Study setting

This study was conducted at the *Hospital de Clínicas* (HC) of Universidade Estadual de Campinas (Unicamp), a university Hospital with over 400 beds situated in the southeastern Brazil. The hospital has a total of 62 ICU beds distributed across five adult ICUs: Coronary Care Unit, Trauma, Transplant, Neurological and General. All the ICU within the hospital were considered in this study. This hospital serves as a reference point for a population exceeding 3.5 million inhabitants. Its mission to revolves around providing public health care of medium and high complexity while concurrently promoting the development of science and education with a commitment of safety, quality, respect, and sustainability [24].

### Study participants

For the recruitment phase, information about the ongoing study was disseminated among 50 eligible nurses in the targeted ICUs. The communication was facilitated through their supervisors and ICU group on a social network site. Inclusion criteria comprised: (1) Being a Registered Nurse (RN); and (2) Possessing a minimum of two years of experience in ICU. This criterion aligns with Benner’s Novice to Expert Model, considering nurses with at least two years of experience to be at the competent level, capable of prioritizing tasks based on their past experiences [25].

The 20 nurses demonstrating interest to participate received a letter providing a detailed overview of the research and information about the primary investigator (ACBSG). They were encouraged to contact the researcher to schedule a meeting.

The study sample ultimately comprised 16 nurses, including both bedside nurses and nurse supervisors. As the training needs may be perceived differently based on the individuals expressing them, it was essential to consult a broad spectrum of people for a comprehensive understanding. The recruitment was interrupted when the saturation criterion, indicating that the data obtained became redundant, was satisfied [26].

### Data collection

Initially, the focus group strategy was chosen as the primary method for data collection. Focus groups, characterized by group interviews emphasizing communication and interaction between research participants serves to explore participant’s knowledge and experiences on a specific topic, delving on perceptions, beliefs, and attitudes [27]. Typically, according to Kitzinger (1995) the ideal group size falls between four and eight participants, fostering rich exchanges and assuring active participation from all individuals [27]. However, resource constraints, particularly the challenge of freeing up more than one nurse during the same shift, compelled us to reduce the number of participants in each group. Consequently, five groups were formed, with two to four nurses in each, totaling 14 nurses. Despite the smaller sample size in the focus groups, as compared to the literature recommendations, we chose to retain this strategy acknowledging its potential advantages, as the detailed and nuanced conversations fostering more meaningful interactions among participants [27]. This decision is aligned with our goal of obtaining qualitative insights rather than seeking generalizability through statistical representation. To both validate and deepen our understanding of the CPD training needs of the ICU nurses, two face-to-face individual interviews were conducted [28]. This decision aimed to create a more intimate setting, providing participants with the opportunity to express personal views in-depth. By combining both methods, our goal was to achieve a comprehensive understanding of the training needs of ICU nurses, capturing the breadth of group dynamics and the depth of individual perspectives.

All the sessions were conducted in a private room within the ICU ensuring utmost privacy and confidentiality with participants only. Each session, lasting up to 30 min, was audiotaped, and led by the primary investigator (ACBSG), a female RN, MSc, PhD candidate (ACBSG) who underwent training provided by the research supervisors for effective data collection.

**Fig. 1:**
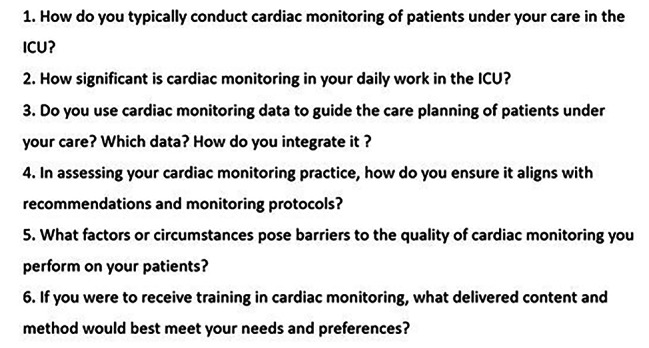
Questions guiding the Focus Groups and Interviews

The focus groups and interviews followed a semi-structured format with questions formulated based on a literature review on the nurses’ experiences and the barriers encountered in cardiac monitoring practice. This question guide was submitted to face validity by two experts in ICU research (Fig. 1) and was centered on the ICU nurses’ daily work [29].

Data collection adhered to key principles of qualitative studies: providing appropriate spaces, ensuring the confidentiality of records, and using guided questions with clear explanations about the study objectives before commencement [30, 31]. A neutral distance was maintained by the primary researcher conducting the interviews to prevent personal opinions from influencing the data collection. Upon conclusion of the individual interview or focus group, nurses provided the following information: age (years), sex (male/female) and length of experience as RN and in ICU (years).

### Data analysis

**Fig. 2:**
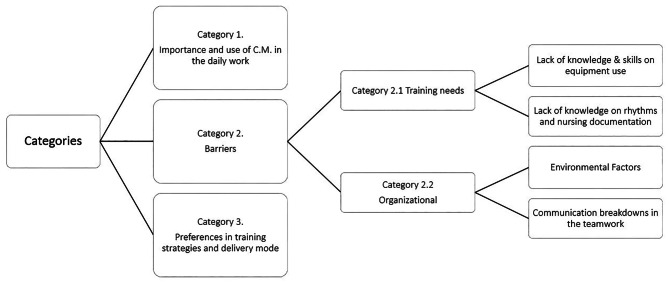
Categories and subcategories emerging from the content analysis

The data underwent a content analysis following a structured sequence: transcription of each recording, fluctuant and iterative reading, unitization, categorization, coding, description, and interpretation. The **confirmability** of data was ensured by the neutrality of the primary investigator conducting data collection making sure that the findings were shaped exclusively by the respondents and not by the researcher bias. Transcripts underwent two reviews by three different researchers, guaranteeing through scrutiny and minimizing potential bias. In adherence to analyst triangulation aimed at enhancing **credibility**, the initial content analysis performed by the primary investigator (ACBSG) was cross-verified by two other researchers (MCG, MEC) [32–33]. To meet the criteria of **dependability**, an external researcher not involved in the initial analysis (JFG) assessed the findings and data interpretation. Finally, to ensure **accuracy** and **reliability**, after the formation of primary codes, the content analysis was scrutinized in light of participants’ comments. Data saturation was achieved revealing key themes that include: Importance and use of cardiac monitoring in the daily work; Barriers categorized into training needs and organizational aspects (each further divided into two subcategories) and Preferences in training strategies and delivery modes (Fig. 2).

## Results

Study participants included registered nurses (RN) (*n* = 13) and nurse supervisors (*n* = 3), predominantly female (94%), with an average age of 37.7 years. On average, participants had 12.9 years of experience as a RN and 7.7 years in the ICU.

In the upcoming section, we delineate the three overarching categories derived from the analysis of interviews and focus groups. The excerpts of participants’ statements presented are freely translated from the original Brazilian Portuguese.

### Category 1: importance and use of cardiac monitoring in the daily work

All nurses acknowledged the importance of cardiac monitoring in their daily work in ICU. Due to the critical condition of ICU patients, nurses perceive cardiac monitoring as a crucial tool for early detection of cardiac rhythm abnormalities and subsequent intervention. Given their continuous presence at bedside, they view themselves as the most suitable professionals for this monitoring.

***Nurse-16****“I believe it (*cardiac monitoring*) is extremely important, it guides interventions and provides insight into the patient’s clinical condition…I think it is important for the nurse to know that because with cardiac monitoring and proper dynamics evaluation, we can even predict complications…”*.

***Nurse-9****“It is important for the critically ill patient in the ICU, that you assess them in real time, there, moment by moment, frequent…”*.

***Nurse-13****“The nurse’s role is fundamental in* cardiac monitoring, *because as we spend most of our time with patients, especially in the ICU. We are the ones who observe the monitor for any morphological changes. We are the ones who initiate the entire team…”*.

However, the majority of nurses stated that they did not adhere to a specific protocol for the practice of cardiac monitoring, often relying on automatic or routine practices. For instance, the lead DII was commonly used for all patients without a clinical judgment of its adequacy.

***Nurse-9****“Here, we use lead D2 in all patients…”*.

### Category 2: barriers

Despite recognizing the significance of cardiac monitoring, nurses highlighted various barriers to its effective implementation which were classified into two subcategories: those related to factors possibly addressed by a CPD and the other ones, related to organizational aspects. Those concerning more specifically training needs were divided in Lack of knowledge & skills in equipment use and, Lack of knowledge on rhythms & documentation. The barriers related to organizational aspects were further divided in Environmental factors and, Communication breakdowns within the teamwork.

### Subcategory 2.1: training needs

### Lack of knowledge & skills on equipment use

Addressing barriers, nurses highlighted lack of knowledge and skills for effectively handling the monitor, particularly when dealing with new equipment. They expressed a lack of formal training during the introduction of new technologies, relying on mutual support to bridge this training gap.

***Nurse-9****“There is no formal training when new equipment arrives, (so) we’re learning a little bit every day.”*

***Nurse-14****“We know there is a lack of knowledge and training in new technologies, and we learn from each other every day.”*

Additionally, nurses emphasized the insufficient knowledge and skills in setting alarms properly. Generally, they rely on default criteria, and if specific adjustments are necessary based on the patient’s condition, physicians are typically responsible for making those changes. Nurses also noted a common practice of adjusting alarm settings to minimize false alarms, thereby reducing their own stress levels. They recognized that this inappropriate use of cardiac monitoring could heighten risks to the patient.

***Nurse-5****“The machines have alarms; but nurses don’t set them, it’s the physicians who do. Most alarms are either in a standard configuration or adjusted to minimize alarm stress in ICU, rather than being tailored to the individual needs of the patients based on their pathophysiology.”*

### Lack of knowledge on rhythms & nursing documentation

Nurses overwhelmingly expressed a lack of knowledge in recognizing various types of rhythm disorders and struggled with proper documentation of these abnormalities. They voiced concerns about the absence of standardized documentation to guide them in improving cardiac monitoring practices. Documentation was typically performed only when significant events or interventions took place. The nurses identified barriers to documentation, including insufficient time and understaffing.

***Nurse-12–****“I struggle to recognize the cardiac arrest rhythms.”*

***Nurse-9*** *“I document cardiac monitoring only when a significant event occurs.”*

***Nurse-12*** *“document only if there is an intervention; if it is a self-resolving alteration, I don’t record anything.”*

***Nurse-14*** *“In my routine, I don’t register anything if there is no noticeable alteration.”*

***Nurse-5****“There is no specific protocol for nursing documentation about cardiac monitoring/ arrhythmias…”*.

### Subcategory 2.2 Organizational

#### Environmental factors

Nurses highlighted problems with equipment maintenance, pointing out that technical issues with the monitor often take a considerable amount of time to be addressed. Additionally, the repaired equipment frequently exhibits limitations in monitoring quality.

***Nurse-2****“Maintenance is logistically time-consuming and most of the time, no cables or spare parts are available.”*

Another environmental factor contributing to challenges was the non-compliance with the existing protocol guiding the local practices in cardiac monitoring. Some nurses have demonstrated a lack of awareness regarding an existing protocol, while others appear not to feel accountable for consulting it.

***Nurse − 1****“We have a protocol, but we don’t worry about implementing it in practice.”*

***Nurse****− 2 “We have an intranet protocol, but we don’t use it, it is just there to say it exists.”*

### Communication breakdowns in the Teamwork

Nurses underlined communication issues within the healthcare team. It was observed that various healthcare professionals informally adjust, set, or change alarms without notifying the nursing team. The absence of an implemented protocol contributes to the use of diverse criteria for alarm settings. Physicians were identified as the primary professionals making these changes, seldom communicating them to the nurses.

***Nurse– 8****“We encounter difficulties communicating with the doctors, so I don’t set alarms, they do. They never discuss it with us.”*

***Nurse − 7****“There is no point in studying protocols if the doctors are the only ones who set the alarms…”*.

### Category 3: preferences in training strategies and delivery modes

Nurses unanimously emphasized the critical need for training in cardiac monitoring, encompassing electrocardiographic interpretation and the identification of rhythm disorders.

***Nurse − 14****“We really need electrocardiographic training, I don’t know how to recognize the different rhythms, I have to be honest.”*

***Nurse − 6****“We have never been trained for this; I didn’t even know there is an AHA cardiac monitoring guideline. It would be great to be trained.”*

***Nurse − 16****“Would be great to know the 2017 AHA guideline, I never heard about it, it would also be nice to know the correct technique.”*

When asked about the preferred mode of intervention delivery for long-term cardiac monitoring training, all nurses stressed the importance of a combination of theoretical and practical activities. However, opinions were divided on the optimal theoretical training strategy. Most mentioned a preference for E-learning strategies combined with face-to-face interventions. Opinions diverged on whether to undertake the training during or outside working hours.

## Discussion

This study aimed to assess the training needs of ICU nurses for the development of a CPD, focusing on implementing standard practices in cardiac monitoring in within a Brazilian university hospital. The identification of training needs involved the elicitation of their experience, understanding perceived barriers and considering preferences in training. The study followed a CPD framework as outlined by Blais & Hallé (2003).

Blais & Hallée (2003) [19] highlight the common practice of proposing training activities before establishing a clear definition of the underlying problem and its etiology, that is, the training needs. Making an analogy of the practical problem to be solved to a “symptom,” they emphasize the importance of understanding the factors contributing to its manifestation. In the context of inadequate cardiac monitoring by ICU nurses, root causes may involve a lack of knowledge or updates, personal barriers like motivation deficiencies, and environmental factors such as unfavorable conditions or outdated equipment.

A comprehensive analysis of the indicator and its underlying factors is crucial not only for identifying training needs but also for recognizing issues that go beyond training solutions. Administrative corrections, often part of the recommended interventions, become apparent through this analysis.

Our data strongly align with Blais & Hallée’s assertions. Despite acknowledging the critical importance of optimal cardiac monitoring for critically ill ICU patients, nurses in this study conceded that the quality of their practice of cardiac monitoring fell short of recommended standards, thus confirming the existence of the problem or “symptom” to be addressed.

Various barriers were identified as contributors to the disparity between the established standards and the actual practice in cardiac monitoring. Most of the barriers were related to training needs.

Nurses strongly emphasized deficiencies in both equipment use and knowledge on rhythms and nursing documentation. In the complex environment of caring for critically ill adult patients within a highly technological setting, where the integration of equipment and technology poses challenges in nurses’ daily responsibilities [34], deficiencies in both equipment use and knowledge of rhythms and nursing documentation become increasingly evident. The ongoing evolution of technology, while bringing numerous benefits, also presents challenges, acting as a barrier to the effective handling and optimal utilization of these tools [8]. Even experienced ICU nurses, without continuous training, may encounter difficulties related to technology, leading to the underutilization of tools, misinterpretation of data, and potentially impacting the quality of person-centered care [5, 34–36]. This emphasizes the crucial role of a CPD in addressing these gaps. In this context, CPD should not only cover fundamental standards of monitoring and their adaptation to specific patient clinical conditions, it should also function as a strategic solution to navigate and overcome the intricate challenges in the technological healthcare landscape.

Still in relation to equipment, our data indicate that nurses are confronted with difficulties on appropriately setting alarms when managing monitors. This task, integral to equipment use, seems to present an additional hurdle for them. Ensuring effective alarm settings is crucial to prevent false or non-actionable alarms, maintaining their primary purpose of promptly detecting abnormalities without disrupting patient care. False alarms triggered in the absence of proper cardiac monitoring can negatively impact patients’ well-being by noise disturbances and delayed staff response times due to alarm fatigue [37–39]. Since 2007, the ECRI Institute has consistently identified alarm-related risks among the top health technology hazards, underscoring the potential dangers associated with medical devices. They advocate for improving alarm settings as a key step in minimizing the likelihood of patient adverse events [40].

To optimize patient safety, alarms should be tailored to the individual’s clinical condition, considering factors such as the presence of arrhythmias or a low heart rate, and also accounting for potential technical issues [41]. The practice of alarm personalization is crucial for minimizing alarm-related risks but requires preparation, knowledge, and continuous training of ICU professionals [6]. A CPD planning must include a multi-method approach to enhance the safety of alarm systems [42–45].

Our findings highlight a prevalent perception of insufficient knowledge among nurses regarding electrocardiographic interpretation and abnormalities identification. This knowledge gap is crucial as ICU patients frequently encounter clinical conditions that may lead to new-onset or worsening cardiac arrhythmias, potentially resulting in hemodynamic impact and life-threatening situations. Consistent with our results, studies indicate a significant challenge in promoting nurses’ knowledge in electrocardiographic interpretation, with the additional difficulty of maintaining this knowledge in daily clinical practice [46]. To address these issues, a CPD should encompass a comprehensive training on electrocardiographic interpretation with a specific focus on its clinical application as well as the integration of a scenario-based learning to simulate real-life cases, allowing nurses to practice identifying and responding to cardiac arrhythmias. Regular updates and refresher courses can ensure continuous learning and proficiency in electrocardiographic interpretation.

Difficulties in the documentation of cardiac monitoring was a point also frequently highlighted by the nurses. Cardiac monitoring documentation involves not only identifying cardiac rhythm but also comprehensively documenting abnormalities, their impact, and interventions. Electrocardiographic readings’ documentation is crucial for accurate diagnosis and effective treatment. The American Heart Association’s (AHA) standards of practice for electrocardiographic monitoring emphasize documenting waveforms on admission or during events, details of arrhythmias, interventions (before, during, after), leads used, outcomes, and abnormalities [6]. Thus, nursing documentation is undeniably critical for ensuring continuity of care and patient safety, as well as for insurance purposes, clinical decision-making, and various other relevant situations. Therefore, it is paramount that a CPD includes training on a standardized procedure of documentation to ensure quality throughout the cardiac monitoring process [37, 47–48].

Regarding their expectations for cardiac monitoring training, in addition to content specifications, nurses emphasize characteristics of a suitable CPD. This includes a learner-centered approach focused on motivation from both personal and employment-related perspectives, incorporating theoretical and practical activities delivered through asynchronous e-learning and hands-on strategies.

Providing training to nurses in a critical activity such as cardiac monitoring in the ICU is paramount, primarily for ensuring the quality and safety of care and improving patient outcomes. Additionally, this training serves as a source of empowerment for nurses, enhancing their clinical competencies and fostering leadership within the interdisciplinary team [49–50].

As a result, to address these issues a CPD based on holistic approach to learning incorporating concrete experiences, reflective observation, abstract conceptualization, and active experimentation seems to be a good avenue for those nurses.

Experiential learning, involving applying knowledge and skills to real-world scenarios could be particularly beneficial for cardiac monitoring, as it allows nurses to directly apply what they learn to the challenges they encounter in their clinical practice [51]. Through concrete experiences allowing reflective observation, abstract conceptualization, and active experimentation a deeper understanding and retention of information can be promoted what is crucial for nurses who need to apply their knowledge quickly and accurately in dynamic healthcare settings [51–52].

A pedagogical strategy promoting active engagement is likely to enhance motivation. As nurses mentioned, they even know the existence of a protocol for the cardiac monitoring but did not feel compelled to use it. Motivated and engaged nurses are more likely to apply their learning in their daily practice [53].

An interesting approach to address these points could be a CPD based on the principles of Kolb’s Theory of Experiential Learning, that is based on expanding on the professional experience. This well-rounded method aligns with the diverse challenges in the knowledge translation of standard cardiac monitoring practice.

Moreover, the principles of Kolb’s Theory of Experiential Learning are aligned with nurses’ expectations. The methods based on this theory can accommodate diverse learning styles, making it suitable for various educational formats. This flexibility allows for a blend of asynchronous e-learning and hands-on strategies, catering to the varied preferences and schedules of nurses.

It is noteworthy that along with training needs, our data indicate that environmental factors served as well as impediments to the implementation of standard practices. Nurses particularly highlighted equipment-related issues such as monitor quality and maintenance, as well as the absence of practice standardization in protocol development and implementation. Another noteworthy concern was the communication within the interdisciplinary team regarding these practices. A CPD, even when tailored to the population’s needs, may prove ineffective in translating knowledge into action if these organizational aspects function as barriers. Addressing these issues requires increased involvement from managers, supervisors, and interdisciplinary collaboration to facilitate the knowledge translation of standard practices in cardiac monitoring in clinical settings [54]. Tackling these organizational aspects in conjunction with a CPD is imperative for a comprehensive and effective approach.

## Conclusion

ICU nurses presented clear and specific training needs related to cardiac monitoring as knowledge, skills, and competencies. Other organizational aspects were also reported as barriers. Addressing these learning needs through targeted CPD aligned with organizational initiatives can contribute to enhancing the quality of cardiac monitoring practices in ICUs.

### Electronic supplementary material

Below is the link to the electronic supplementary material.

Supplementary Material 1

## Data Availability

Not applicable. The datasets used and/or analyzed during the current study are available from the corresponding author on reasonable request.

## Abbreviations

AHA: American Heart Association
CPD: Continuous Professional Development
ICU: Intensive Care Units
KT: Knowledge Translation
